# A single-nucleotide polymorphism in the proximal promoter region of the apolipoprotein M gene is associated with dyslipidaemia but not increased coronary artery diseases in Chinese populations

**DOI:** 10.1186/1476-511X-12-184

**Published:** 2013-12-16

**Authors:** Bing Cao, Yi Zhou Ye, Jun Rui, Ming Qiu Li, Wei Wang, Liu Yan Wei, Guo Qing Jiao

**Affiliations:** 1 Department of Surgery, Affiliated Taixing People’s Hospital, Yangzhou Medical University, Taixing City, Jiangsu Province, P. R. China; 2 Department of Cardiovascular Surgery, Affiliated Shanghai 1st People’s Hospital, Shanghai Jiaotong University, Shanghai, P. R China; 3 Department of Cardiovascular Surgery, Affiliated Wuxi People’s Hospital, Nanjing Medical University, Wuxi City, Jiangsu Province, P. R. China; 4 Department of Cardiovascular Surgery, National Center for Cardiovascular Disease, Beijing, P. R. China

**Keywords:** apoM, Coronary artery disease, Genetic polymorphism, Risk factor

## Abstract

**Background:**

It has been reported that rs940494 and rs805296 SNPs of apolipoprotein M (apoM) gene may confer the risk in the development of type 2 diabetes (T2D) and coronary artery disease (CAD) in the Han Chinese. However, a recent study demonstrated that rs805297 polymorphism is significantly associated with reduced total high density lipoprotein (HDL) levels in rheumatoid arthritis patients. But the relationship between rs805297 SNP and CAD has not been explored. The aim of the present study was to elucidate whether the rs805297 mutant allele is implicated in CAD and links to changes in blood lipid levels in these patients.

**Methods:**

Three hundred CAD patients and three hundred and twelve non-CAD patients were subjected in the present study. All subjects were confirmed by the angiography. Plasma concentrations of apoM were semi-quantitatively determined by dot-blotting analysis, and total serum lipid levels were quantified using an automated RA-1000 (Technician, USA). The genotyping of rs805297 of apoM was analyzed by polymerase chain reaction–restriction fragment length polymorphism (PCR–RFLP).

**Results:**

Genotype and allele frequencies were not significant (P = 0.5798 and 0.3834, respectively) between cases and controls. Compared with the wild-type C/C genotype, carriers of the C/A and A/A genotypes did not have an increased risk of CAD, as determined by multiple logistic regression analysis, after adjustment for age, sex, BMI, history of smoking, hypertension and hypercholesterolemia. (CA, odds ratio = 0.49, 95% confidence interval 0.15–1.87, *P* = 0.462; AA, odds ratio = 0.51, 95% confidence interval 0.13–1.68, *P* = 0.534). The plasma concentration levels of apoM did not differ significantly among carriers of the three genotypes between two groups. Lastly, control subjects with A/A genotypes had lower total levels of HDL cholesterol than did those with C/C genotypes.

**Conclusions:**

The results presented here suggest that the rs805297 SNP is not associated with an increased risk of developing CAD, although it does independently correlate with dyslipidaemia in Han Chinese individuals.

## Introduction

Coronary artery disease (CAD) is one of the most common causes of death globally, including in China. CAD is a complex multi-factorial and polygenic disorder that is thought to result from the interactions of an individual's genetic background and several environmental factors [1,2].

Apolipoprotein M (apoM) is predominantly present in HDL, and to a lesser extent in chylomicrons, very low density lipoprotein (VLDL) and low-density lipoprotein (LDL) [3]. It plays an important role in reverse cholesterol transport [4]. The apoM gene is proposed to play a protective role against the development of CAD [5]. Recent studies suggest that several single nucleotide polymorphisms (SNPs), including rs805297, rs940494 and rs805296 occur in the Han Chinese population. Rs940494 and rs805296 have been identified to associate with Type 2 diabetes or CAD [6-8]. Rs805297 SNP is significantly associated with reduced total HDL levels in rheumatoid arthritis patients [9]. It is well known that reduced high-density lipoprotein (HDL) cholesterol levels is one of risk factors of CAD, but the relationship between rs805297 and CAD has not been explored.

The objective of the present study was to investigate whether there is an association between a common SNP in the apoM gene, namely the C-1065A polymorphism, and the risk of CAD in a Chinese population. We chose to evaluate this SNP in particular because it is located in the apoM promoter region, and changes in apoM expression levels may increase the risk of CAD or other complex diseases where lipid levels are pathologically altered.

## Results

The clinical parameters of study subjects in the CAD and control groups are described in Table 1. There were no significant differences in gender, age or several conventional risk factors between groups, including habitual smoking and TC; however, CAD patients demonstrated significant differences in hypertension, TG, HDL-C and LDL-C concentrations (Table 1).

**Table 1:** Characteristics of the total study population

	***CAD patients*** **(*n*** = ***300*)**	***Control subjects*** **(*n*** **=*312*)**	***P*-*Value***
***Age*** **(*years*)**	62.3 ± 10.1	60.8 ± 11.2	0.321
***Sex*** **(*M*/*F*)**	194/106	183/129	0.126
***Habitual smoking* (%)**	42.7	40.1	0.427
***BMI*** **(*kg*/*m***^***2***^**)**	25.1 ± 3.09	23.9 ± 3.2	0.068
***Hypertension***			
***Yes***	131*	79	0.000
***No***	169	233	
***TC*** **(*mmol*/*L*)**	4.51 ± 1.31	4.47 ± 1.25	0.072
***TG*** **(*mmol*/*L*)**	2.18 ±0.93*	1.84 ± 1.02	0.013
***HDL*-*C*** **(*mmol*/*L*)**	1.08 ±0.34*	1.25 ± 0.42	0.032
***LDL*-*C*** **(*mmol*/*L*)**	2.27 ± 0.97*	2.39 ± 0.78	0.000

Data are presented as mean ± SD for age, body mass index (*BMI*), total cholesterol (*TC*), triglycerides (*TG*), low-density lipoprotein cholesterol (*LDL*-*C*), high-density lipoprotein cholesterol (*HDL*-*C*), coronary artery disease (*CAD*). *p < 0.05 vs control group.

**Figure 1:**
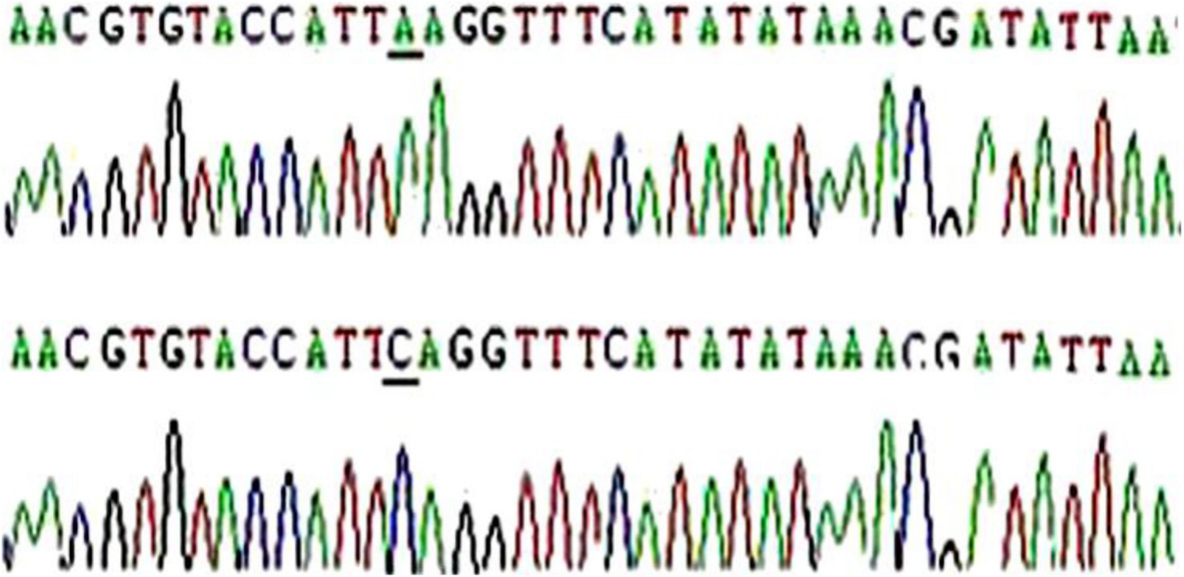
**DNA sequencing of the polymorphic region (C-A transition at nucleotide -1065) in the proximal promoter region of the apoM gene revealed that rs805297(C > A) of apoM gene is valid in Han Chinese.** This nucleotide change results in a Tsp509I restriction site.

**Figure 2:**
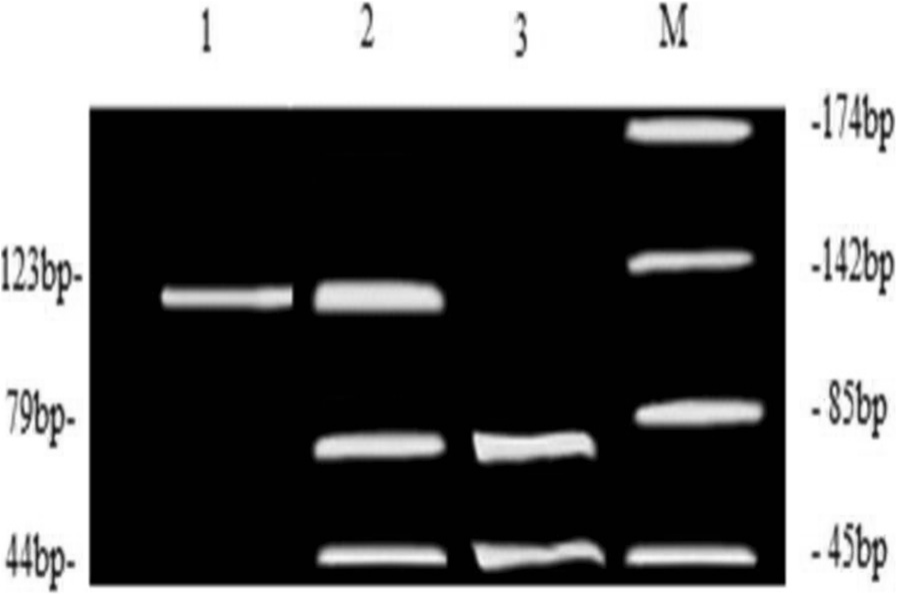
**Restriction fragment length polymorphism analysis results for the apoM rs805297(C > A) locus.** Products were separated on a 4% low molecular weight agarose gel and stained with ethidium bromide. Lane M, ladder of molecular size markers; lane 1, CC homozygote; lane 2, CA heterozygote; lane 3, AA homozygote.

The validity of the rs805297 SNP of the apoM gene in the Han Chinese population was determined by DNA sequencing (Figure 1). Additionally, the polymorphism was confirmed by PCR-RFLP (Figure 2).

Genotype distributions and allele frequencies of rs805297 SNP in CAD cases and controls are summarized in Table 2. Chi square analysis demonstrates the allele frequencies to be in Hardy-Weinberg equilibrium. There were no significant differences identified between CAD and controls for either genotype distributions (χ2 = 1.090, df = 2, *P* = 0.5798) or allele frequencies (χ2 = 0.7597, df = 2, *P* = 0.3834). Compared with the wild-type C/C genotype, carriers of the C/A and A/A genotypes did not have an increased risk of CAD, as determined by multiple logistic regression analysis, after adjustment for age, sex, BMI, history of smoking, hypertension and hypercholesterolemia (Table 3). Our analysis did not detect any effect of the apoM genotype on the likelihood of having CAD.

**Table 2:** Distribution of genotypes between CAD patients and control subjects

**Genotypes**	**CAD patients (n = 300)**	**Control subjects (n = 312)**	**P**-**Value**
C-1065A			
CC	154 (51.3%)	147 (47.1%)	
CA	137 (45.7%)	155 (49.3%)	
AA	9 (3.0%)	10 (3.6%)	0.5798^#^
C allele	445 (74.2%)	449 (72.0%)	
A allele	155 (25.8%)	175 (28.0%)	0.3834^##^

^#^Differences of apoM genotypes between *CAD* patients and controls were tested in 2 × 3 contingency tables.

^##^Differences of allele frequencies between *CAD* patients and controls were tested in 2 × 2 contingency tables.

*CAD*, coronary artery disease.

**Table 3:** Logistic regression analysis for the association between apoM genotypes and the risk of coronary artery disease

**Genotypes**	**OR**	**95% CI**	**P-Value**
CC	0.41	0.09—1.38	0.452
CA	0.54	0.12—1.63	0.364
AA	0.47	0.16—1.72	0.743

*ApoM* genotypes were set as categorical variables (homozygote with major allele = 0; heterozygote = 1; homozygote with minor allele = 2) model. Odds Ratio (*OR*) and 95% confidence interval (*CI*), as well as the *P* value, were calculated with adjustment for age, sex, *BMI*, history of smoking, hypertension and hypercholesterolemia by logistic regression.

**Figure 3:**
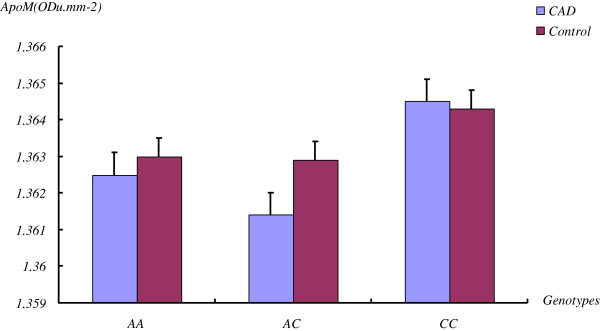
**Plasma concentrations of apoM among different genotypes of rs805297 of apoM for both CAD patients and controls.** The average plasma concentrations of apoM in CAD patients of AA, CA, CC genotypes were 1.3625 ± 0.1053 ODu.mm^-2^, 1.3614 ± 0.1271 ODu.mm^-2^ and1.3645 ± 0.1432 ODu.mm^-2^, and in controls were 1.3630 ± 0.1127 ODu.mm^-2^, 1.3629 ± 0.1132 ODu.mm^-2^ and1.3643 ± 0.1021 ODu.mm^-2^, respectively. There were no significant differences in the plasma concentrations of apoM among these three genotypes in these subjects by Wilcoxon signed ranked test (*p* ≥ 0.05).

We further analyzed the impact of the rs805297 SNP on serum lipid concentrations in both CAD patients and controls. Controls carrying the AA polymorphism demonstrated significantly lower HDL-C concentrations compared to wild-type CC subjects in control groups (Table 4). It was reported that rs805297 SNP affects the levels of apoM mRNA expression in rheumatoid arthritis patients. Luciferase reporter assay suggested that the C to A substitution at the promoter region of apoM affects the transcription activity in vitro [9]. We investigated the plasma concentration of the apoM protein to determine whether it changes as a function of the genotypes. The average plasma concentrations of apoM in CAD patients with AA, CA, CC genotypes were 1.3625 ± 0.1053 ODu.mm^-2^, 1.3614 ± 0.1271 ODu.mm^-2^ and 1.3645 ± 0.1432 ODu.mm^-2^, and in controls were 1.3630 ± 0.1127 ODu.mm^-2^, 1.3629 ± 0.1132 ODu.mm^-2^ and 1.3643 ± 0.1021 ODu.mm^-2^, respectively. There were no significant differences in the plasma concentrations of apoM between two groups by Wilcoxon signed ranked test (***p*** > 0.05) (Figure 3).

**Table 4:** Lipid profile values (mmol/L) depending on apoM genotypes

***Genotype***								
**CAD patients (n = 300)**					**Control subjects (n = 312)**			
	CC	CA	AA	P-Value	CC	CA	AA	P-Value
TC	4.52 ± 1.21	4.48 ± 1.45	4.46 ± 1.31	0.694	4.48 ± 1.27	4.45 ± 1.19	4.43 ± 1.06	0.482
TG	2.19 ±1.01	2.18 ±1.22	2.21 ±0.97	0.837	1.93 ± 1.12	1.81 ± 1.05	1.88 ± 0.97	0.631
HDL-C	1.09 ± 0.23	1.11 ± 0.38	1.05 ± 0.35	0.153	1.27 ± 0.42	1.24 ± 0.37	1.07 ± 0.39*	0.043
LDL-C	2.25 ± 1.07	2.28 ± 1.13	2.31 ± 0.98	0.147	2.39 ± 0.75	2.38 ± 0.83	2.33 ± 0.68	0.298

Data are expressed as mean ± SD. *CAD*, coronary artery disease; *TG*, triglycerides; *TC*, total cholesterol; *LDL*-*C*, low-density lipoprotein cholesterol; *HDL*-*C*, high-density lipoprotein cholesterol. *P* value for effect of the *ApoM* genotypes on plasma lipid concentration within *CAD* patients and controls by ANOVA. *p < 0.05 vs control group.

## Discussion

The pathophysiology of atherosclerosis is multifactorial with environmental, metabolic and genetic components influencing etiology [10]. A large body of literature has established a panel of traditional candidate genes implicated in cardiovascular disease, such as angiotensin-converting enzyme (ACE) and angiotensinogen (AGT). Variants in these traditional genes are known to be correlated with coronary artery disease (CAD). Recent studies have identified new loci to the growing list of the candidate genes, including myocyte enhancer factor 2 (MEF2A) and phosphodiesterase 4 (PDE4D) [11]. ApoM is a novel potential candidate gene that may be protective against the development of CAD [5].

In present case–control study, we sought to determine whether rs805297 SNP of the apoM gene alters susceptibility to CAD. First, we compared genotype distributions and allele frequencies of the apoM gene between CAD and control groups and then performed a logistic regression with adjustments for age, sex, BMI, history of smoking, hypertension and hypercholesterolemia. Our data identified no relationship between CAD and the apoM genotype, supporting that rs805297 SNP is not a risk factor for genetic susceptibility to CAD.

Previous studies have reported associations between rs805297 and rs940494 SNPs of apoM with CAD in Chinese populations [7,8]. Contrarily, our study did not detect any differences in genotype distributions and allele frequencies of rs805297 SNP of apoM gene between CAD and control groups in our Han Chinese subjects. This may be attributed to different allele frequencies of apoM rs805297 in our study compared to previous studies [6], which may be due to differences in age, sex, race, geographic location or population sample size. Our study is limited by it’s case–control design; future prospective studies that include haplotype analysis may overcome some of these limitations [12].

It is thought that apoM plays a role in lipid metabolism; however, the biological mechanisms of this protein are not fully understood. Previous studies have identified associations between apoM and plasma lipoproteins, demonstrating correlations with size, charge and composition [13]. Plasma apoM is strongly correlated with total cholesterol in healthy individuals [14], with apoM concentrations roughly corresponding to 1/50th of the apoA-I plasma concentration [15]. This prompted us to investigate the impact of the C-1065A polymorphism on the plasma lipid profile. Consistent with a recent report [9], our study found no significant differences between rs805297 SNP and atherogenic lipids (TC, TG, LDL-C). We found no significant differences in HDL between CAD patients with A/A genotypes and those with C/C or C/A genotypes, although control subjects with A/A genotypes had lower levels of HDL than those with C/C or A/A genotypes. It is possible that statin treatments for hyperlipidemia in CAD patients modulated the lipid parameters.

The apoM gene is located in histocompatibility complex III (HMC-III) region on chromosome 6. The rs805297 SNP site is localized to the promoter, which may affect gene transcription and concomitant protein levels in the blood to underlie its disease association. This hypothesis is supported by previous literature indicating that the C allele of rs805296 SNP may increase promoter activity and confer the risk susceptibility to type I diabetes [16]. Additionally, Hae-Jin Hu and colleagues suggested that rs805297 polymorphism in the apoM promoter may decrease its transcriptional activity and thus alter HDL-C concentrations to ultimately affect RA susceptibility [17]. In light of these previous findings, we evaluated the impact of rs805297 polymorphism on plasma concentration of apoM. Based on non-significant differences in plasma apoM concentrations, our data suggests that the mutant promoter does not affect apoM gene expression. It is possible that this polymorphism is functionally silent which many explain our negative finding. In the present study, we used a semi-quantitative method to estimate serum apoM levels; therefore, future studies should apply more precise measures to determine whether this finding is replicable.

## Conclusions

The data in this present study support that the rs805297 SNP is not associated with increased susceptibility to CAD. Our data indicates that this polymorphism is associated with dyslipidemia in healthy controls in the Han Chinese populations.

## Material and methods

### Subjects

Subjects recruited for this study consisted of 300 CAD patients (194 males and 106 females with a mean age of 62.3 ± 10.1 years) and 312 unrelated individuals enrolled as control subjects (183 males and 129 females with a mean age of 60.8 ± 11.2 years). To be enrolled in the CAD group, patients, had to have at least 50% stenosis, determined angiographically, in at least one of the major segments of coronary arteries, i.e., the right coronary artery, left circumflex, or left anterior descending arteries. CAD patients suffering from acute coronary syndrome or undergoing previous cardiovascular events such as angioplasty or CABG or stenting were not enrolled in CAD group. Controls were required to have a negative coronary artery angiography so as to exclude CAD. History of conventional risk factors for CAD, including habitual cigarette smoking (persons with smoking habit smoke more than 1 cigarette per day averagely), hypertension (systolic blood pressure ≥160 mm Hg and/or diastolic blood pressure ≥95 mmHg), diabetes mellitus (A diagnosis of diabetes is confirmed if there are symptoms of diabetes and a plasma glucose level of at least 11.1 mmol/L, a fasting plasma glucose level of at least 7 mmol/L; or a two-hour plasma glucose level of at least 11.1 mmol/L during an oral glucose tolerance test.), or hypercholesterolemia (TC ≥ 5.7 mmol/L) were obtained from the subjects or from their medical records. Exclusion criteria for both the CAD patients and control individuals included familial hypercholesterolemia, diabetes mellitus, cancer, renal disease, and any other chronic illnesses. All subjects in both the CAD and control groups are Han Chinese from Jiangsu province or Shanghai, China. The research was in agreement with Helsinki declaration. The research was prospectively reviewed and approved by a duly constituted ethics committee at Nanjing Medical University. Written informed consent was obtained from each subject before collecting blood samples. A medical history and a peripheral venous blood specimen were obtained from all participants; part of this blood sample was analyzed for plasma levels of fasting glucose, triglycerides (TG), total cholesterol (TC), HDL–cholesterol (HDL-C) and LDL–cholesterol (LDL-C), all of which were measured using an automated RA-1000 (Technician, USA).

### Genotyping of the human apoM gene

Five milliliters (5 ml) of venous blood was collected from each subject into 50 mmol/L disodium EDTA as an anticoagulant, and genomic DNA was extracted from peripheral blood leukocytes, as described previously [18]. The apoM polymorphism was analyzed by polymerase chain reaction–restriction fragment length polymorphism (PCR–RFLP). DNA was amplified using the following primers: forward primer, 5′-GCTTTGCAAACATTACTATTCAT-3′, reverse primer, 5′-ATTGGCAAATCATCAATCTTATA-3′.^6^ Amplification reactions were performed in a total volume of 50 μl containing: 1 μg of genomic DNA, 12.5 pmol of each primer, 2.5 mmol/L each of dCTP, dTTP, dGTP, and dATP, 2 U of Taq DNA polymerase (Promega, USA), 50 mmol/L KCl, 1.5 mmol/L MgCl_2_. 0.1% Triton X-100 and 10 mmol/L Tris–HCl (pH 9.0 at 25°C). The thermocycling procedure consisted of an initial denaturation at 94°C for 5 min; 35 cycles of denaturation (94°C for 30 s), annealing (48°C for 30 s), and extension (72°C for 30 s); and a final extension at 72°C for 5 min. PCR products were first subject to restriction digest by Tsp509I for 16 h at 65°C; the reaction was performed in a total volume of 20 μl containing 1 μg of PCR product; 5 U of restriction endonuclease Tsp509I (New England Biolabs, USA) and 2 μl of restriction enzyme buffer. Genotypes were then used to detect the presence of *apoM* polymorphisms on a 4% low molecular weight agarose gel electrophoresis system (Promega, USA) and visualized by ethidium bromide staining. The Cys→Ala substitution creates a Tsp509I restriction site in the rs805297 polymorphism of apoM. PCR products with an Ala allele were digested into two fragments (79 and 44 bp), whereas the PCR product with the Cys allele could not be cut by Tsp509I. To confirm detection of the C-1065A polymorphism of the apoM gene by PCR–RFLP, PCR products were purified with Centricon-100 filtration devices (Amicon, Beverly, MA, USA) and sequenced directly with a fluorescence-based automated DNA sequencer (Applied Biosystems, Foster City, CA, USA). All experiments were performed in duplicate in order to obtain precise assessments of genotype for each subject.

### Measurement of plasma apoM concentration

Plasma apoM concentration was estimated by dot-blot analysis using monoclonal rabbit anti-human apoM antibody (ABNOVA). Briefly, 10 μl of plasma was diluted 1:20 in Tris–HCl buffer and 5 μl of the diluted samples was applied to a membrane in triplicate (Hybond-C, Amersham Life Science). Membranes were quenched in Tris–HCl buffer with 4% Tween and 3% bovine serum albumin for 2 hours, followed by sequential incubations with monoclonal rabbit anti-human apoM antibody (1:1000 in Tris–HCl buffer) overnight at 4°C, and then alkaline phosphatase (AP)-conjugated secondary antibody for 2 hours at room temperature. AP levels were measured with a commercial visualization system following the manufacturer’s instructions (Dako). The recombinant ApoM protein (ABNOVA) and ddH_2_O were used as positive and negative controls for evaluation, respectively. The relative amounts of apoM in diluted plasma samples were quantified by a scanner using the Quantity One software (Version 4.2.1, Bio- Rad Laboratories) and presented as adjusted volume (ODu.mm^-2^). Each experiment was repeated at least in triplicate.

### Statistical analysis

Clinical data from CAD patients and control subjects was compared by the unpaired Student's t test or the Mann–Whitney U test. Allele frequencies were estimated by the gene-counting method, and the χ2 test was used to identify significant variations from the Hardy–Weinberg equilibrium. We employed two models to assess differences in allele frequencies and genotypes between CAD patients and controls: comparing allele frequencies in 2 × 2 contingency tables or genotypes in 2 × 3 contingency tables. We also performed multivariable logistic regression analysis to adjust for risk factors. CAD was assigned as a dependent variable, and the independent variables included age, body mass index, gender (male = 0, female = 1), smoking status (0 = nonsmoker, 1 = smoker), metabolic variables (0 = no history of hypertension, or no hypercholesterolemia; 1 = positive history), and apoM genotype. All genotypes were set as categorical variables (homozygote with major allele = 0; heterozygote = 1; homozygote with minor allele = 2). The OR and 95% CI were also calculated. Plasma apoM levels for the different genotypes were compared between two groups by Wilcoxon signed ranked test, and the significance threshold was set to *P* = 0.05. Statistical analysis was done using the SPSS statistical software package version 11.5.

## Competing interests

The authors declare that they have no competing interests.

## Authors’ contributions

All authors contributed equally in the study. CB, YYZ and JGQ participated in the project design. All authors read and approved the final manuscript.

## Authors’ information

*Department of Surgery, Affiliated Taixing People’s Hospital, Yangzhou Medical University, Taixing 225400, People's Republic of China. ^#^Department of Cardiovascular Surgery, Affiliated Shanghai 1st People’s Hospital, Shanghai Jiaotong University, Shanghai 210008, People's Republic of China. †Department of Cardiovascular Surgery, Affiliated Wuxi People’s Hospital, Nanjing Medical University, Qingyang Road 299, Wuxi City, Jiangsu Province 214023, China. * †Department of Cardiovascular Surgery, National Center for Cardiovascular Disease, Beijing 200000, People's Republic of China. Co-First Author: Yi Zhou Ye.
